# Advances in research on the relationship between thymoquinone and pancreatic cancer

**DOI:** 10.3389/fonc.2022.1092020

**Published:** 2023-01-04

**Authors:** Zhanxue Zhao, Linxun Liu, Shuai Li, Xiaofan Hou, Jinyu Yang

**Affiliations:** ^1^ Suzhou Medical College of Soochow University, Suzhou, Jiangsu, China; ^2^ Department of General Surgery, Qinghai Provincial People’s Hospital, Xining, Qinghai, China; ^3^ Department of Clinical Pharmacy, Affiliated Hospital of Qinghai University, Xining, Qinghai, China; ^4^ Graduate school, Qinghai University, Xining, Qinghai, China

**Keywords:** pancreatic neoplasm, pharmaceutical preparation, drug resistance, traditional medicine, thymoquinone

## Abstract

Pancreatic cancer has one of the worst prognoses among the most common cancers in the world. Its characteristics include a high rate of metastasis and chemotherapeutic resistance, which present major challenges to the medical community. The potential anticancer effects of thymoquinone (TQ), which is the main bioactive compound of the black seeds of the *Nigella sativa* plant, have recently received widespread attention for their potential use in treating pancreatic cancer. TQ can inhibit cell proliferation, promote cancer cell apoptosis, inhibit cell invasion and metastasis, enhance chemotherapeutic sensitivity, inhibit angiogenesis, and exert anti-inflammatory effects. These anticancer effects predominantly involve the nuclear factor (NF)-κB, phosphoinositide 3 kinase (PI3K)/Akt, Notch, transforming growth factor (TGF)-β, c-Jun N-terminal kinase (JNK), and p38 mitogen-activated protein kinase (MAPK) signaling pathways as well as the regulation of the cell cycle, matrix metallopeptidase (MMP)-9 expression, and pyruvate kinase isozyme type M2 (PKM2) activity. TQ regulates the occurrence and development of pancreatic cancer at multiple levels and through multiple targets that communicate with each other. In this review, we summarize and discuss the analogs and carriers of TQ that have been developed in recent years. Given its multilevel anticancer effects, TQ may become a new therapeutic drug for treating pancreatic cancer in the future. This review presents a brief introduction to the research that has been conducted on TQ in relation to pancreatic cancer to provide a theoretical basis for future studies on the topic.

## Introduction

Pancreatic cancer has one of the worst prognoses of all major cancers worldwide. According to the latest global cancer statistics in 2020, pancreatic cancer accounts for 2.6% of new global cancers, and the mortality rate accounts for 4.7% of all cancer deaths (1). According to data released by the American Cancer Association in 2022, the mortality rate is almost the same as the incidence rate and the 5-year overall survival rate is only approximately 8%. Pancreatic cancer ranks 8th and 10th for the number of new cancer cases in the United States for females and males, respectively, and the mortality rate is the 3rd highest among all malignant tumors (2). In China, the incidence rate of pancreatic cancer is increasing every year, having overtaken bladder cancer to become the 7th most common tumor in men and exhibiting the 7th highest cancer-related mortality rate among the Chinese population (3). The high metastatic rate, low chemosensitivity, and high mortality of pancreatic cancer have long been major challenges in the medical community.

**Figure 1:**
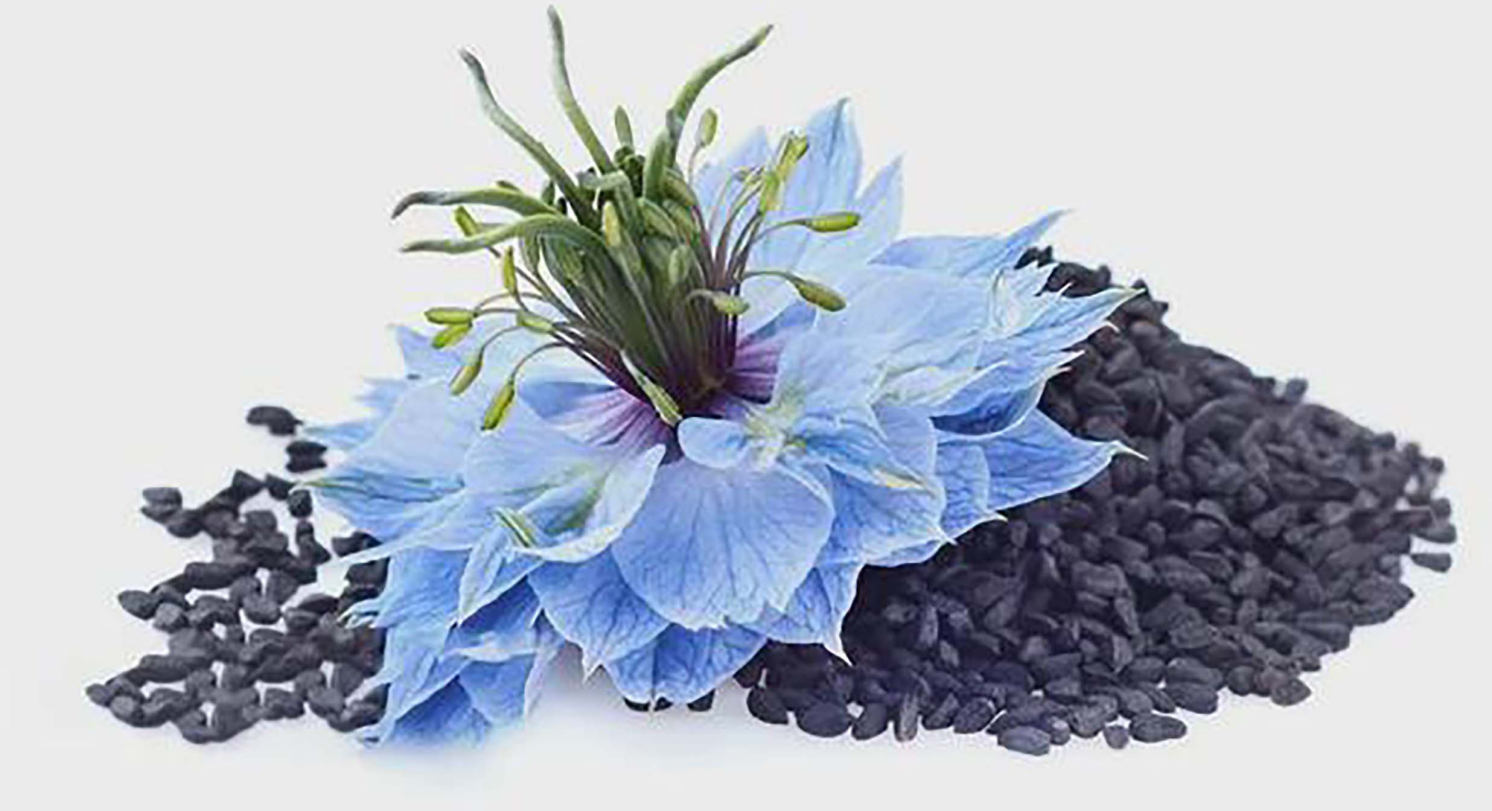
Photograph of black seeds from the *Nigella sativa* plant.

*Nigella sativa* seed oil (**Figure 1**), which is derived from the *Nigella sativa* plant, is a traditional medicinal material used in Arab countries, South and Southeast Asia, the Mediterranean, China, and various African countries. It is traditionally used to prevent and treat various diseases and disorders, including bronchial asthma, eczema, hypertension, diabetes, rheumatism, cough, bronchitis, headache, fever, influenza, neurological disorders, gastrointestinal problems, cancer, and various inflammatory diseases (4). According to the US Food and Drug Administration, *Nigella sativa* seed oil is categorized as “Generally Recognized as Safe” (5). The components of *Nigella sativa* seeds include fixed oil (22–38%), volatile oil (0.40–0.45%), alkaloids (0.01%), amino acids and proteins (21–31%), carbohydrates (25–40%), saponins (0.013%), vitamins (1–4%), minerals (3.7–7%), and terpenoids, p-isocyanate, limonene, thiamine, niacin, and folic acid with different compositions (**Table 1**) (6). The main fatty acids of fixed oil are linoleic acid (64.6%) and palmitic acid (20.4%). The identified amino acids mainly include cysteine, methionine, glutamic acid, aspartic acid, and arginine. The minerals mainly include iron, copper, zinc, phosphorus, and calcium (7). Moreover, the volatile oil of black seeds contains approximately 18.4–24.0% of thymoquinone (TQ) as the main bioactive ingredient.

**Table 1:** Ingredients of nigella sativa seed.

ingredients	percentage	reference
carbohydrates	25%–40%	(6, 7)
fixed oil (inoleic acid, palmitic acid)	22%–38%	
amino acids (cysteine, methionine, glutamic acid, aspartic acid, arginine)	21%–31%	
minerals (iron, copper, zinc, phosphorus, calcium)	3.7%–7%	
vitamins	1%–4%	
volatile oil (thymoquinone)	0.40%–0.45%	
saponins	0.01%	
alkaloids	0.01%	
others (terpenoids, p-isocyanate, limonene, thiamine, niacin, folic acid)	3%-4%	

## Characteristics of TQ

**Figure 2:**
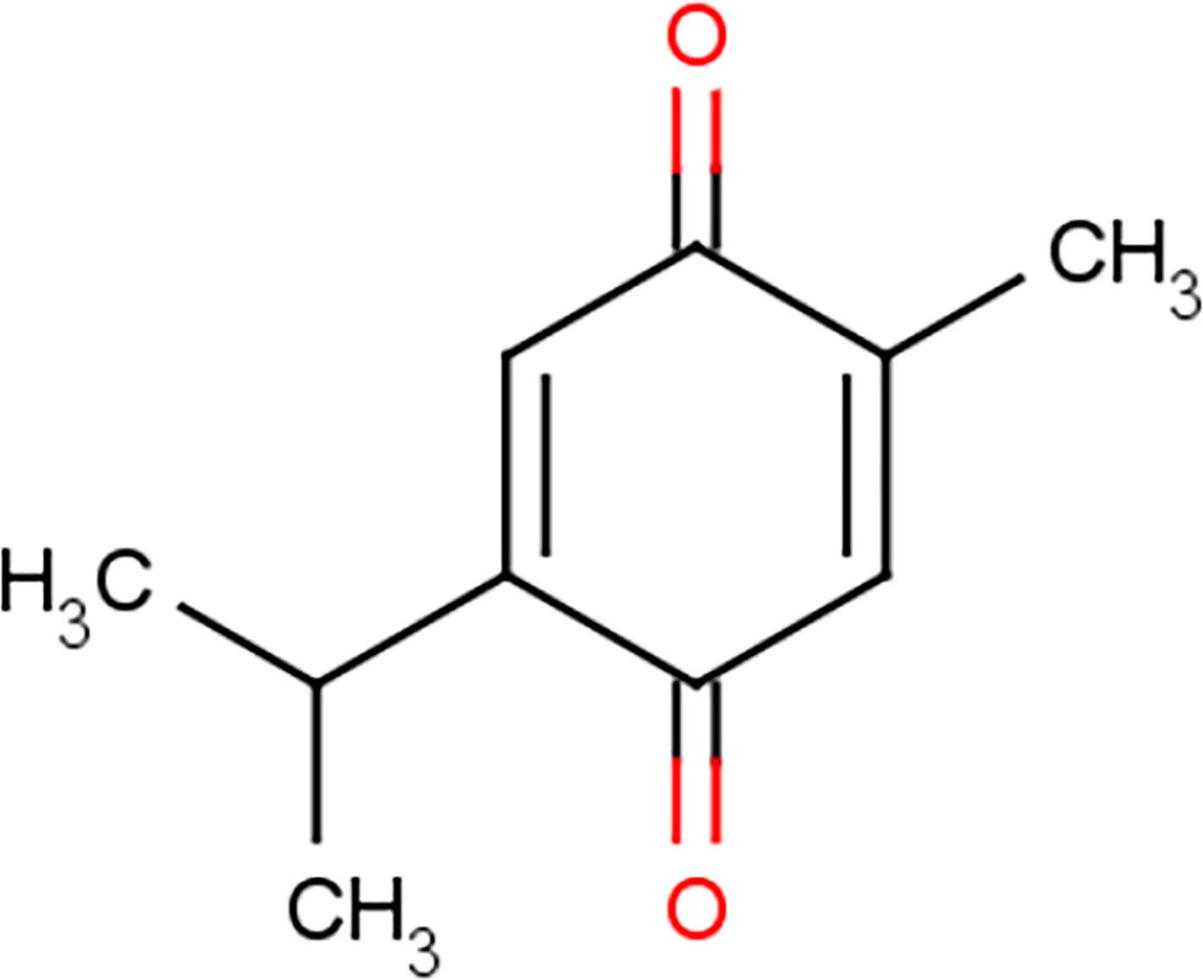
Chemical structure of thymoquinone.

TQ was first separated in 1960 and is a yellow sediment that is slightly soluble in water (8). Chemically, TQ has the molecular formula C10H12O2 and a molecular weight of 164.204 g/mol (9). It has a basic quinone structure consisting of a para-substituted dione conjugated to a benzene ring to which a methyl and isopropyl side chain groups are added at positions 2 and 5, respectively (**Figure 2**) (10, 11). Because of its special chemical structure, TQ has characteristics such as high fat solubility, poor hydrophobicity, and low bioavailability (12). The bioavailability of TQ has been documented ~58% with a lag time of ~23 min (13). Analysis of the interface of TQ with blood constituents reflects the effect on its bioavailability, metabolism, distribution, and emission. The results recommended that covalent attaching of TQ to bovine serum albumin guides in losing the TQ antitumor action against checked tumor cells; in contrast, the TQ antitumor activity has been not affected while TQ is bound with alpha-1 acid glycoprotein. This property is determined by the special chemical structure of thymoquinonez (14). TQ can be degraded significantly when exposed to light for a short period of time (15, 16). It is unstable under alkaline conditions, and its stability can be reduced with an increase in pH (17). When administered orally, TQ has a fast elimination rate and a slow absorption rate (13, 18). Therefore, it has not been largely studied. However, with the increase in the application of nanoparticle technology, the pharmacological effects of TQ began to garner more attention.

Researchers have found that TQ exhibits a wide range of pharmacological activities, including antioxidant, anti-inflammatory, antifibrotic, antidiabetic, antihistamine, anticancer, antimicrobial, and anticonvulsive effects (5, 19, 20). The antitumoral effects of TQ mainly include antiproliferation, apoptosis induction, anti-metastasis, cell cycle arrest, reactive oxygen species (ROS), and other regulatory mechanisms (21–23). Moreover, while TQ inhibits the growth of many tumor cells, it exerts no harmful effects on normal cells, which makes TQ a promising anticancer drug (24). In animal experiments, TQ did not exhibit toxicity when injected (5 mg/kg, mice; 12.5 mg/kg, rats) or administered orally (100 mg/kg, rats) (19, 20). The median lethal dose (LD50) in mice was 104.7 mg/kg after intraperitoneal injections of TQ and 870.9 mg/kg after oral ingestion. The LD50 in rats was 57.5 mg/kg after intraperitoneal injections and 794.3 mg/kg after oral ingestion (25). TQ has further exhibited antitumoral effects when administered in small concentrations of less than 10 mg/kg (25, 26).

Recently, research on TQ and pancreatic cancer has attracted substantial attention. In this review, we briefly describe the current state of global research on TQ and pancreatic cancer to provide a point of reference for further exploration (**Table 2**).

**Table 2:** Anticancer mechanism of TQ against pancreatic cancer.

reagent	experimental model	mechanisms	effects	references
*in vitro* studies				
TQ	PANC-1	none	↓proliferation	(27)
TQ	FG/COLO357, CD18/HPAF	↓MUC4, ↑JNK, ↑p38 MAPK, ↑TGF-β, ↑ROS, ↑FAK	↑apoptosis, ↓migration	(28)
TQ	PANC-1	↑ROS, ↓MMP- 9	↑apoptosis, ↓migration	(29)
TQ	MiaPaCa-2, ASPC-1	↑p53, ↑p21, ↑Bax, ↓Bcl-2, ↑Bax/Bcl-2, ↑H4, ↓HDAC, ↓G0/G1	↑apoptosis,↓proliferation	(30)
TQ	PANC-1,	↓NF-κB, ↓MMP- 9, ↓CD34	↓migration,↓invasion	(31)
TQ	BxPC-3	↓FAK/PI3K/AKt, ↓F-actin	↓migration,↓invasion	(32)
TQ and juglone	MIA PaCa-2	none	↑apoptosis	(33)
TQ and GEM	PANC-1, AsPC-1, BxPC-3	↓NF-κB,↓Bcl-2, ↓Bcl-xl, ↓XIAP, ↑Bax, ↑cytochrome c, ↑caspase-3, ↑caspase-9, ↓Notch1/PTEN, ↓PI3K/AKT/mTOR, ↓G0/G1	↑apoptosis, ↑chemosensitivity	(34)
TQ and GEM	BxPC-3	↓Bcl-2,↓Bcl-cl, ↓XIAP, ↓survivin, ↑caspase-3, ↑caspase-9	↑apoptosis,↓proliferation,↑chemosensitivity	(35)
TQ and GEM	HPAC, BxPC-3, Panc-1, MDA Panc-28, COLO 357, L3.6pl	↓NF-κB	↑apoptosis,↑chemosensitivity	(36)
TQ, GEM, oxaliplatin	BxPC-3, HPAC	↓survivin, ↓Bcl-xl, ↓ XIAP, ↑caspase-3, ↑caspase-9, ↑PARP, ↓COX-2, PGE2 ↓, G0/G1, ↓S	↑apoptosis,↓proliferation,↑chemosensitivity	(37)
TQ and GEM	PANC-1, MIA PaCa-2	↓PKM2	↑apoptosis,↓proliferation,↑chemosensitivity	(38)
TQ and GEM	PANC-1, MIA PaCa-2,	↓procaspase3, ↓PARP, ↓PKM2	↑apoptosis,↑chemosensitivity	(39)
TQ and GEM	PANC-1	↓procaspase3	↑apoptosis,↑chemosensitivity	(40)
TQ and GEM	BxPC-3	none	↑apoptosis,↓proliferation,↑chemosensitivity	(41)
TQ	HS766T	↓MCP-1, ↓NF-κB,↓TNF-α	↑anti-inflammatory	(42)
TQ	PANC-1	↓Ki-67, ↓CD34, ↓VEGF	↓angiogenesis	(43)
in vivo studies				
TQ	mice	↓NF-κB, ↓MMP- 9, ↓CD34	↓metastasis	(31)
TQ and GEM	mice	↓XIAP, ↓MMP-9	↓growth, ↓metastasis,↑chemosensitivity	(44)
TQ, GEM, oxaliplatin	mice	↓survivin, ↓Bcl-xl, ↓ XIAP, ↑caspase-3, ↑caspase-9, ↑PARP, ↓COX-2, PGE2 ↓, G0/G1, ↓S	↓weights of tumors, ↓local invasion, ↓lymph node metastasis	(37)
TQ	mice	↓EPCs, ↓VEGF	↓angiogenesis	(43)

TQ, thymoquinone; GEM, gemcitabine; MUC4, mucin 4; JNK, c-jun NH2 terminal kinases; p38 MAPK, p38 mitogen-activated protein kinase; TGF-β, transforming growth factor-β; ROS, reactive oxygen species; FAK, focal adhesion kinase; Bax, Bcl-2-associated X protein; Bcl-2, B cell leukemia/lymphoma-2; COX-2, cyclooxygenase 2; PGE2, prostaglandin E2; HDAC, histone deacetylase; NF-κB, nuclear factor kappa-B; MMP- 9, matrix metallopeptidase 9; VEGF, vascular endothelial growth factor; XIAP, X-linked inhibitor of apoptosis protein; Akt, phosphorylated protein kinase B; PI3K, phosphoinositide 3 kinase; Notch 1, neurogenic gene notch homolog protein 1; PTEN, phosphorase and tensin homolog; mTOR, mammalian target of rapamycin; PKM2, pyruvate kinase isozyme type M2; PARP, poly ADP-ribose polymerase; MCP-1, monocyte chemoattractant protein-1; TNF-α, tumor necrosis factor-α; EPCs, vascular endothelial progenitor cells; ↑, increase or activate; ↓, decrease or inhibit.

## TQ inhibits proliferation and promotes apoptosis in pancreatic cancer cells

As early as 2006, researchers confirmed that TQ can restrict the proliferation of the pancreatic cancer cell line PANC-1 in a dose-dependent manner (27). Torres proposed the following four potential apoptotic mechanisms of TQ in pancreatic cancer (28). First, TQ can downregulate the expression of mucin 4 (MUC4) through the proteasome pathway, which leads to the activation of c-jun NH2 terminal kinases (JNK) and p38 mitogen-activated protein kinase (p38 MAPK) pathways in pancreatic cancer cells, thereby inducing apoptosis. Second, TQ induces pancreatic cancer cells to secrete transforming growth factor-β (TGF-β), which in turn activates the TGF-β pathway and downregulates MUC4, thereby inducing the apoptosis of pancreatic cancer cells. Third, TQ may activate the JNK pathway by stimulating the production of ROS in pancreatic cancer cells, thereby causing the sensitivity of the cancer cells to Fas-mediated apoptosis. In addition, Narayanan et al. confirmed that TQ can inhibit the viability of the PANC-1 cell line and promote its apoptosis, which may be related to the production of ROS (29). The fourth proposed mechanism is that TQ inhibits the migration of cancer cells through the focal adhesion kinase (FAK) pathway. A decrease in MUC4 expression is related to an increase in apoptosis, decrease in motility, and reduced migration of pancreatic cancer cells, whereas the Fas-mediated apoptosis of pancreatic cancer cells is related to the initiation of the JNK and p38 MAPK pathways. Therefore, the downregulation of MUC4- and TQ-induced apoptosis are not isolated events but are induced by the complex interactions between the different signaling pathways in which MUC4 plays a major role. In addition, Relles et al. confirmed that in MiaPaCa-2 and ASPC-1 of pancreatic ductal adenocarcinoma cell lines, TQ inhibits cell proliferation, inhibits cell viability, causes cell cycle arrest in the G0/G1 phases, and induces apoptosis in a dose-dependent manner. TQ can also upregulate p53, induce p21 expression in a p53-independent manner, induce the expression of the Bcl-2-associated X protein (Bax), downregulate the expression of B cell leukemia/lymphoma-2 (Bcl-2), and increase the Bax/Bcl-2 ratio. In addition, TQ mediates the post-translational modification of histone H4 acetylation, changes its epigenetic state, inhibits the expression of histone deacetylase (HDAC), and induces the pro-apoptotic signaling pathway (30, 45).

**Figure 3:**
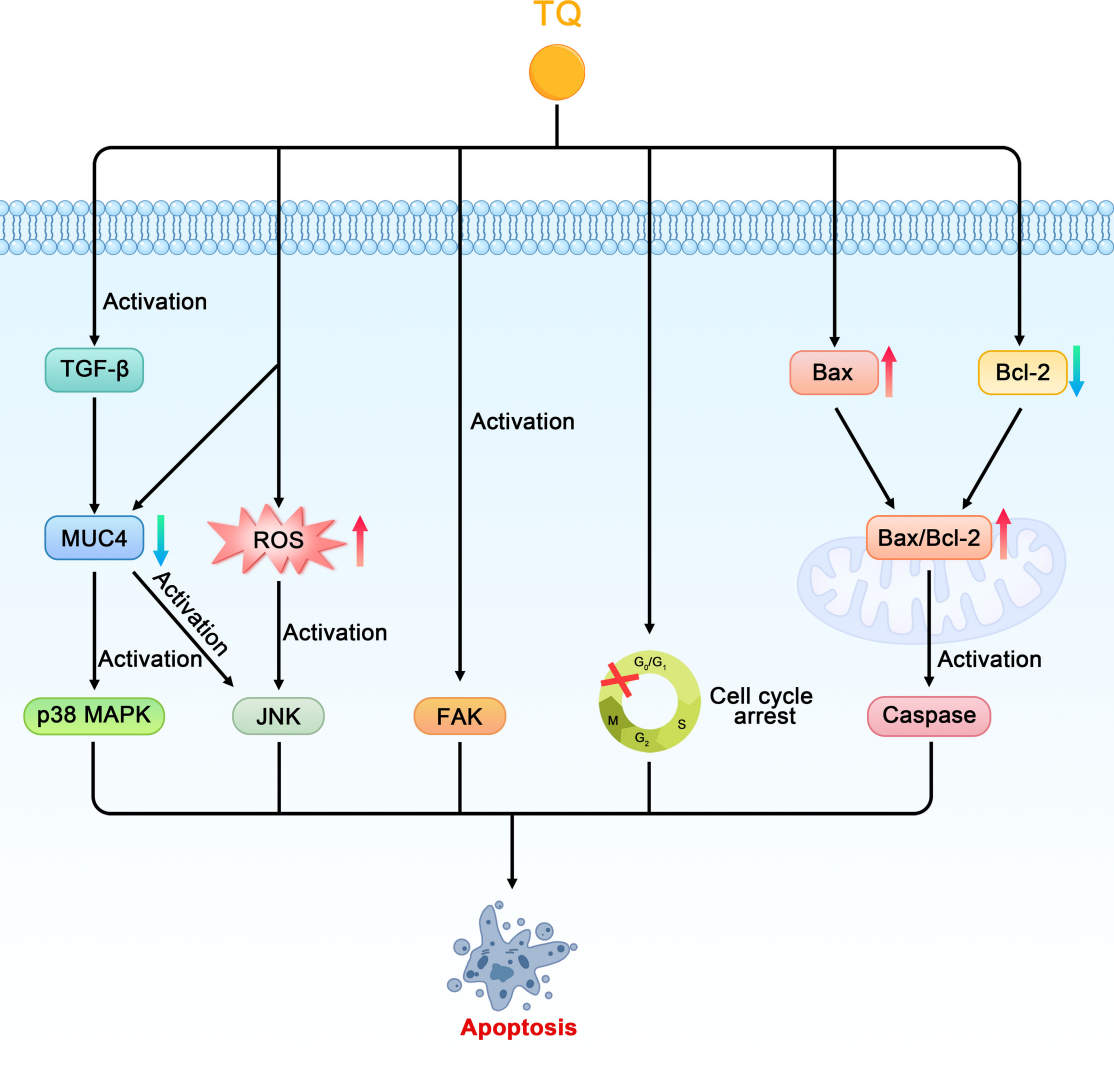
Schematic diagram of the role of thymoquinone in inhibiting cell proliferation and promoting apoptosis in pancreatic cancer (TQ, thymoquinone; MUC4, mucin 4; JNK, c-jun NH2 terminal kinases; p38 MAPK, p38 mitogen-activated protein kinase; TGF-β, transforming growth factor-β; ROS, reactive oxygen species; FAK, focal adhesion kinase; Bax, Bcl-2-associated X protein; Bcl-2, B cell leukemia/lymphoma-2).

Mu et al. further found that TQ downregulates the anti-apoptotic proteins Bcl-2 and Bcl-xl, upregulates the pro-apoptotic protein Bax, induces the release of cytochrome c from the mitochondria of cells from the PANC-1, AsPC-1, and BxPC-3 cell lines, and activates the cysteine-containing aspartate specific protein hydrolase family in a dose-dependent manner, which results in an increase in the cleavage of the active components of caspase-3 and -9 and cell apoptosis (34). Another study found that the toxic effects of juglone and TQ on the MIA PaCa-2 pancreatic cancer cell line changed according to the concentration of both factors. At cell concentrations of 10%, 20%, or 50%, the researchers observed a moderate antagonistic relationship between juglone and TQ. At cell concentrations of 75% and 90%, however, the effect was almost additive. A moderate synergistic effect was observed only at a cell concentration of 95% (**Figure 3**) (33).

## TQ inhibits cell invasion and metastasis in pancreatic cancer

When studying the relationship between TQ and the invasion and metastasis of pancreatic cancer, Wu et al. found that TQ could inhibit the migration and invasion of the pancreatic cancer cell line PANC-1 in a concentration-dependent manner and downregulate nuclear factor kappa-B (NF-κB) and matrix metallopeptidase 9 (MMP- 9). In addition, TQ could significantly inhibit the metastasis of pancreatic cancer in tumor-bearing mice and downregulate the positive expression of CD34, NF-κB, and MMP-9 in pancreatic tumors (31). Narayanan et al. also confirmed that TQ inhibits the migration of PANC-1 cells by reducing the level of MMP-9 (29). MMPs play a key role in promoting biological behaviors such as tumor cell migration, extracellular matrix degradation, and tumor distant metastasis. Type-IV collagenase MMP-9 plays an important role in degrading extracellular matrix proteins and can regulate the vascular endothelial growth factor (VEGF), thereby affecting the formation, invasion, and metastasis of tumor blood vessels. NF-κB, which comprises a family of transcription factor proteins, can regulate the MMP-9 gene at the transcriptional level and directly affect the expression of the MMP-9 protein. In conclusion, TQ can downregulate NF-κB and MMP-9 and their interaction to inhibit the metastasis of pancreatic cancer both *in vitro* and *in vivo*.

**Figure 4:**
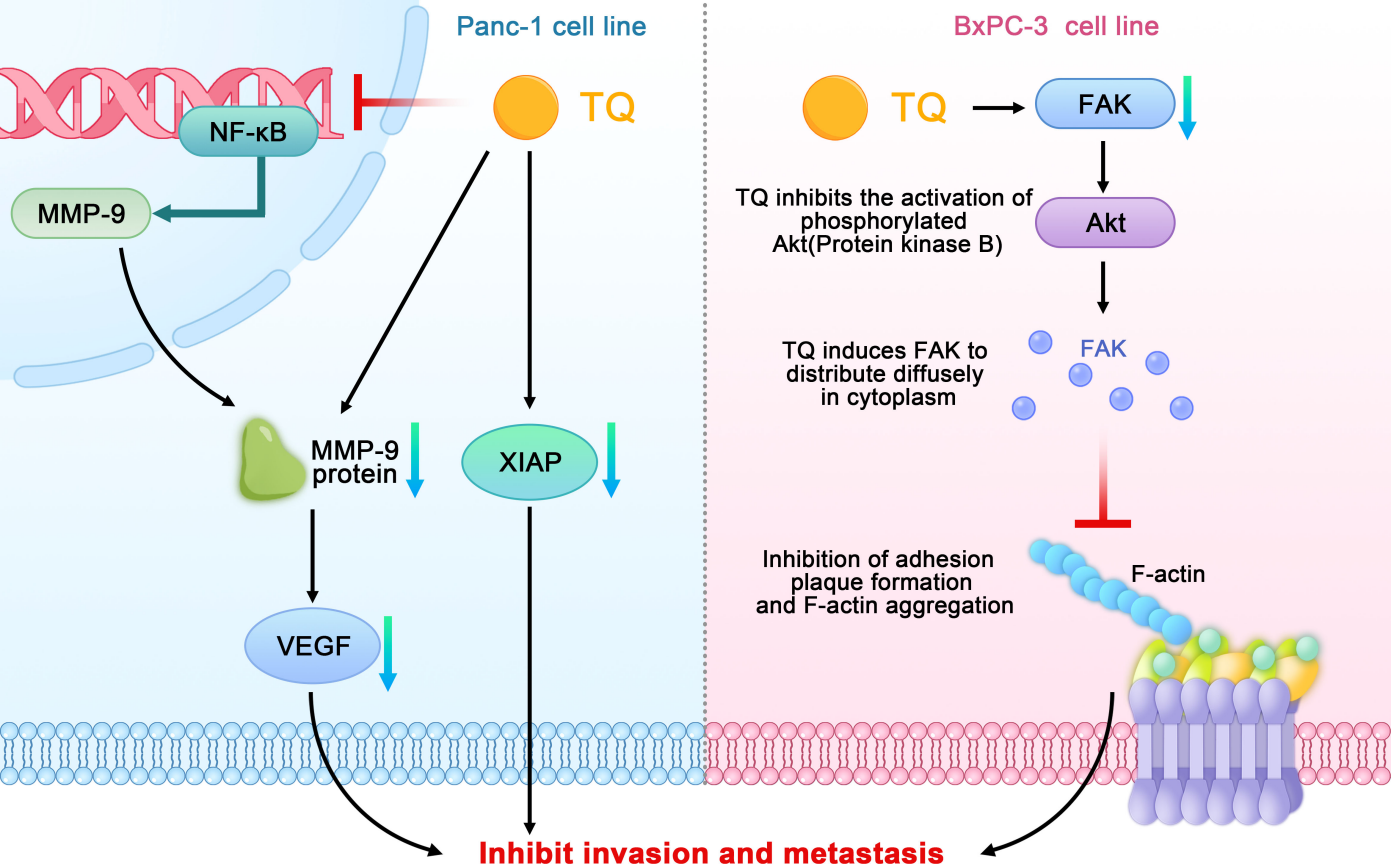
Schematic diagram of the role of thymoquinone in inhibiting the invasion and metastasis of pancreatic cancer (TQ, thymoquinone; FAK, focal adhesion kinase; NF-κB, nuclear factor kappa-B; MMP- 9, matrix metallopeptidase 9; VEGF, vascular endothelial growth factor; XIAP, X-linked inhibitor of apoptosis protein; Akt, phosphorylated protein kinase B).

Wang further found that TQ could inhibit the growth and metastasis of orthotopic transplanted pancreatic cancer tumors in nude mice; this mechanism may be related to the inhibition of the X-linked inhibitor of apoptosis protein (XIAP) and MMP-9 expression (44). Moreover, Mu et al. found that TQ can significantly downregulate the expression of FAK in the pancreatic cancer cell line BxPC-3, inhibit the activation of phosphorylated protein kinase B (Akt), induce the diffusion of FAK in the cytoplasm, and inhibit the formation of focal adhesion and aggregation of F-actin. By inhibiting the signal transduction and kinase activity of the FAK/phosphoinositide 3 kinase (PI3K)/Akt pathway, TQ can inhibit the *in vitro* movement and invasion of pancreatic cancer cells in a concentration-dependent manner (**Figure 4**) (32).

## TQ increases chemosensitivity

The induction of apoptosis and loss of cell viability are the two main mechanisms by which traditional chemotherapeutic drugs kill cancer cells. Gemcitabine (GEM), 2′,2′-difluorodeoxycytidine, is a nucleoside analogue that inhibits DNA synthesis by inhibition of DNA polymerase and ribonucleotide reductase, resulting in cancer apoptosis (46). Unfortunately,GEM-based chemotherapeutic drugs have limited impact on pancreatic cancer due to their dose-limiting toxicity to normal tissues and increased chemoresistance. In an attempt to solve the problem of GEM resistance, Mu et al. found that pretreatment with TQ significantly enhanced apoptosis and growth inhibition by GEM in pancreatic cancer (34, 35). The possible mechanisms were as follows. First, TQ may have enhanced the sensitivity of the pancreatic cancer cells to GEM by inhibiting the neurogenic gene Notch homolog protein 1/phosphorase and tensin homolog (Notch1/PTEN) pathway. A decrease in the activated Notch1 and PTEN protein levels can predict prognoses and chemotherapeutic resistance.GEM can induce the upregulation of Notch1 in pancreatic cancer cells and increase the expression of the Notch intracellular domain (NICD) (47). The TQ pretreatment significantly reduced the upregulation of Notch1 and the Notch intracellular domain induced by GEM; it also restored the PTEN protein that had been inhibited by GEM. The second proposed mechanism was that the PI3K/Akt/mammalian target of rapamycin (mTOR) signaling pathway is involved in all of the signal regulation that is related to the survival and chemoresistance of pancreatic cancer cells. The activation of this pathway and expression of the downstream effector molecule S6 ribosomal protein can enhance chemosensitivity and promote apoptosis in pancreatic cancer cells However, TQ pretreatment significantly attenuates the phosphorylation of mTOR, S6, and upstream Akt caused by GEM. The third proposed mechanism was that TQ can enhance GEM-induced antitumoral activity *in vivo* through NF-κB and its downstream molecules. NF-κB is one reason for the characteristic resistance of pancreatic tumor cells to the apoptotic effects of chemotherapy drugs. Thus, XIAP and survivin, which are members of the family of apoptosis inhibitory proteins that are regulated by NF-κB, are regarded as therapeutic targets because of their involvement in tumor cells, especially in chemotherapeutic resistance, cell proliferation, and angiogenesis. Researchers previously found that, compared with GEM alone, the DNA-binding activity of NF-κB and structural phosphorylation of p65 were significantly reduced in tumor-bearing mice treated with TQ and GEM. In addition, TQ can significantly affect the downstream molecules of NF-κB by downregulating Bcl-2, Bcl-cl, XIAP, and survivin and upregulating caspase-3 and -9 activities related to apoptosis. Wang also found that TQ combined with GEM could significantly inhibit the expression of XIAP and MMP-9 in pancreatic cancer tissues, thereby affecting the growth and metastasis of tumors (44). In other study, TQ pretreatment of pancreatic cancer cells (HPAC, BxPC-3, Panc-1 and MDA Panc-28, COLO 357, and L3.6pl) before GEM can downregulated NF-κB (36). In the fourth proposed mechanism, TQ can achieve stronger apoptotic efficacy than GEM alone by increasing the arrest of the cell cycle in the G1 phase. In addition, compared with GEM or TQ alone, a combined treatment of TQ and GEM has been found to significantly reduce the tumor weights of tumor-bearing mice without causing a serious increase in toxicity depending on weight loss.

Consistent with the above findings, Banerjee et al. found that TQ combined with gemcitabine or oxaliplatin can produce greater antitumoral activity. These results were related to the downregulation of NF-κB activity and its downstream proteins, such as survivin, Bcl-xl, and XIAP. In addition, TQ treatment has been found to lead to the appearance of cleaved caspase-3, -9, and the active components of poly ADP ribose polymerase (PARP) in pancreatic cancer cells, activate the upstream events of the caspase cascade, release cytochrome c, and induce apoptosis through the mitochondria pathway. TQ can also inhibit the expression of cyclooxygenase 2 (COX-2) and the accumulation of prostaglandin E2 (PGE2) caused by COX-2. COX-2 plays an important role in inhibiting apoptosis and enhancing cell growth and angiogenesis in pancreatic cancer; therefore, it is considered a target for drug development. The same study also found that TQ combined with gemcitabine could increase the cell population in the G0–G1 phase, whereas TQ combined with oxaliplatin increased cell cycle arrest in the S phase and decreased the proportion of cells in the G2–M phase. Furthermore, the combination of TQ and gemcitabine or oxaliplatin significantly reduced the weights of the tumors in tumor-bearing mice and inhibited local invasion and lymph node metastasis in pancreatic cancer (37).

**Figure 5:**
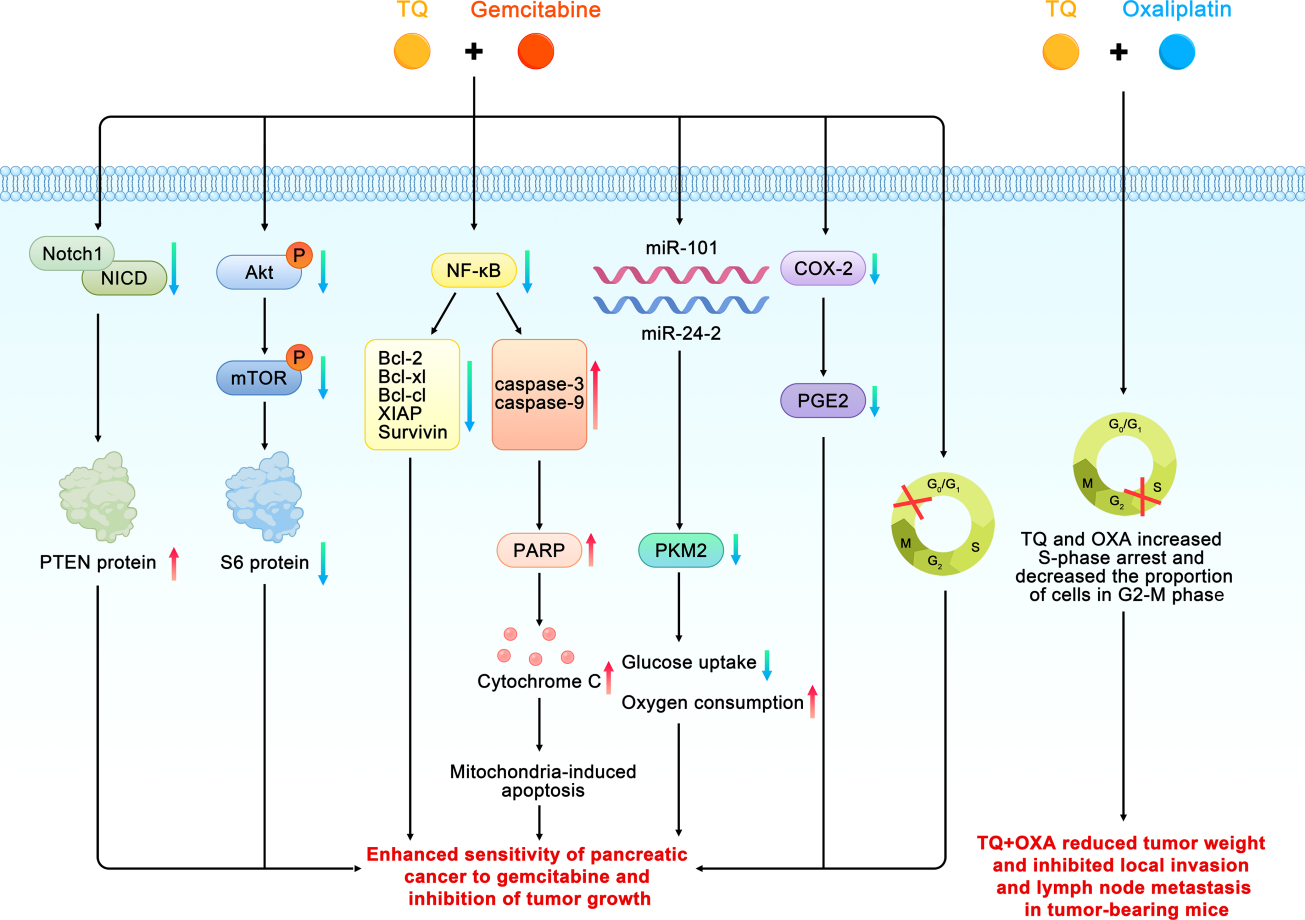
Schematic diagram of the role of thymoquinone in enhancing the chemosensitivity of pancreatic cancer (TQ, thymoquinone; GEM, gemcitabine; Bcl, B cell leukemia/lymphoma; Akt, phosphorylated protein kinase B;Notch 1, neurogenic gene notch homolog protein 1; NICD, Notch intracellular domain; PTEN, phosphorase and tensin homolog; mTOR, mammalian target of rapamycin; PKM2, pyruvate kinase isozyme type M2; PARP, poly ADP-ribose polymerase; COX-2, cyclooxygenase 2; PGE2, prostaglandin E2).

A study by Pandita and coworkers confirmed that compared with GEM alone, the combined application of TQ and GEM could reduce the survival rate of pancreatic cancer cells by more than 50%. Similarly, in a pancreatic cancer xenograft model, the combined application of TQ and GEM had a higher efficiency (approximately 80%) on the impact of tumor weight. Combined drugs have also been found to reduce the expression of pyruvate kinase isozyme type M2 (PKM2) in pancreatic cancer cells. PKM2 is the key enzyme of the Warburg effect in pancreatic cancer, which promotes glucose uptake and reduces oxygen consumption, thereby ensuring the growth of pancreatic cancer cells (38). Some scholars have conducted preliminary research on the relationship between TQ, small molecule RNA, and GEM, and found that the cytotoxicity and apoptotic potential of low-dose GEM play a synergistic role in GEM-sensitive and drug-resistant cell lines through the transfection of miR-101 and miR-24-2 or the combined application of TQ. The synergistic mechanisms of these two microRNAs and TQ in cancer cell lines involve an increase in procaspase3 and poly ADP-ribose polymerase (PARP) cleavage and a decrease in PKM2 activity (39). In addition, the combination effect of TQ and GEM and miR-24–2 in PANC-1 pancreatic cancer cell lines could promote apoptosis through down-regulation of Pro-caspase-3 (40). Wu et al. also found that TQ can effectively enhance the inhibitory effect of GEM on the proliferation of pancreatic cancer cells, where the synergistic effect mainly involves inducing apoptosis (**Figure 5**) (41).

## Anti-inflammatory effects of TQ

**Figure 6:**
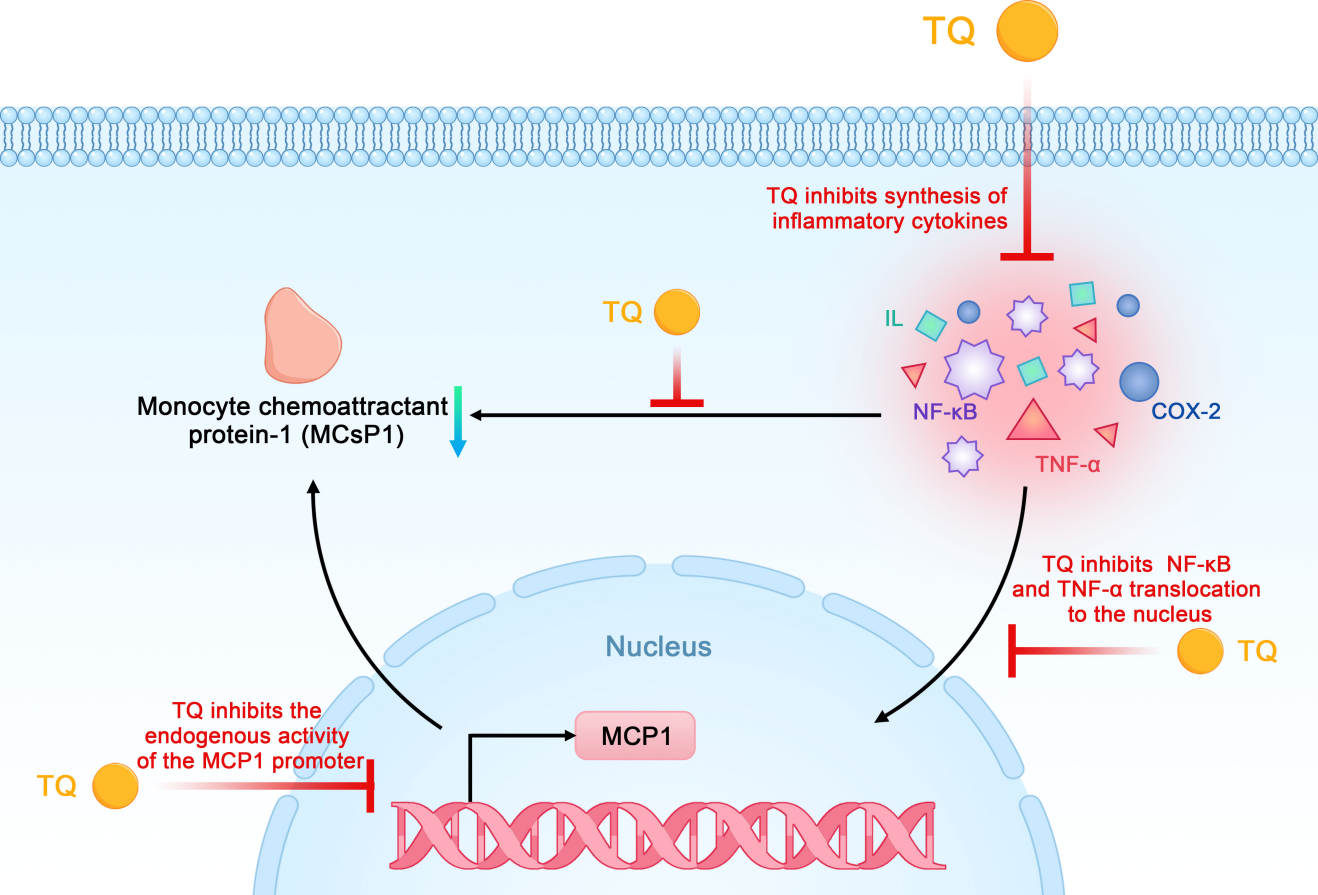
Schematic diagram of the anti-inflammatory effects of thymoquinone on pancreatic cancer (TQ, thymoquinone; COX-2, cyclooxygenase 2; NF-κB, nuclear factor kappa-B; MCP-1, monocyte chemoattractant protein-1; TNF-α, tumor necrosis factor-α).

TQ reduces the synthesis of inflammatory cytokines such as monocyte chemoattractant protein-1 (MCP-1), tumor necrosis factor-α (TNF-α), interleukins, and COX-2 in pancreatic ductal cell carcinoma in a dose- and time-dependent manner. Previous studies have shown that MCP-1 can promote the recruitment of monocytes or macrophages, whereas TQ typically reduces the expression of MCP-1 by inhibiting the endogenous activity of its promoter. NF-κB and TNF-α are involved in cancer progression and are released by immune cells or other stromal cells in tumors, which leads to the production of MCP-1 and greater recruitment of monocytes. TQ inhibits these factors from exerting biological effects by inhibiting the activation of inflammatory factors mediated by NF-κB and TNF-α as well as their transport from the cytoplasm to the nucleus in pancreatic cancer cells (42, 48). The strong anti-inflammatory effects of TQ on NF-κB, TNF-α, and other inflammatory mediators highlight its potential as a preventive and therapeutic strategy for pancreatic cancer (**Figure 6**).

## TQ inhibits angiogenesis

**Figure 7:**
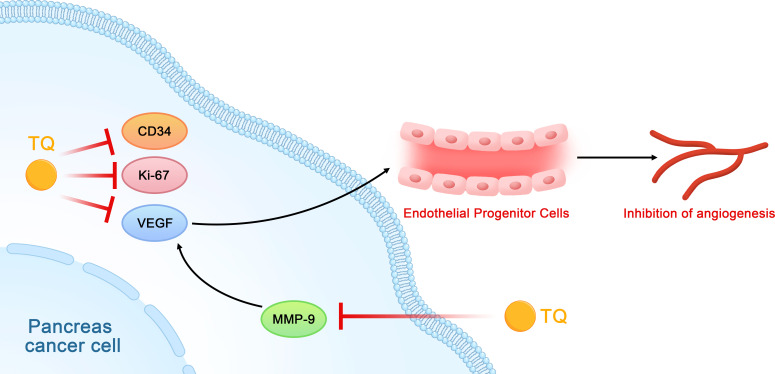
Schematic diagram of the role of thymoquinone in inhibiting the angiogenesis of pancreatic cancer (TQ, thymoquinone; VEGF, vascular endothelial growth factor; EPCs, vascular endothelial progenitor cells).

Studies on the relationship between TQ and tumor angiogenesis have shown that TQ may inhibit the formation of tubules of vascular endothelial progenitor cells (EPCs) *in vitro* by inhibiting the expression of Ki-67, CD34, and VEGF in pancreatic cancer cells and tissues, which then affects angiogenesis in pancreatic cancer (43). Other studies have shown that TQ can downregulate the expression of MMP-9 in pancreatic cancer cells. Since MMP-9 can also regulate VEGF, this downregulation further affects tumor angiogenesis (**Figure 7**) (31, 44).

## Antioxidation and pro-oxidation of TQ

TQ can exert a wide range of antioxidant effects (49). ROS are produced in cells during normal cellular respiration and in response to xenobiotics. As the most important molecule in the oxidative stress response, ROS are vigorously reactive and can oxidatively damage cellular components and alter their functions (50). Oxidative stress is the cause of changes in cell function and structure, including DNA mutations, which lead to the occurrence of cancer. However, studies have not yet found whether TQ can inhibit ROS production in pancreatic cancer *via* its antioxidant effects. However, TQ may stimulate the production of ROS to activate the JNK pathway in pancreatic cancer cells, thereby causing the sensitivity of the pancreatic cancer cells to Fas-mediated apoptosis (28). In addition, TQ can inhibit the activity of PANC-1 cells and promote their apoptosis by inducing ROS production, which has also been confirmed by other researchers (29).

## TQ analogs and carriers

Through various strategies, Banerjee et al. synthesized 3-aminothymoquinone as an initial component, then synthesized its derivatives ATQTHB and ATQTFB with 2,3,4-trihydroxybenzaldehyde and 2,3,4-trifluorobenzaldehyde, respectively. Compared with TQ, these TQ derivatives show improved binding efficiency when binding to the active sites of COX-2. They also exhibited a superior inhibitory effect on tumor cells. The TQ analogs TQ-4A1, TQ-5A1, and TQ-2G are more effective than the parental TQ in the apoptosis of pancreatic cancer cells and can downregulate the DNA-binding effect of NF-κB, inhibit the activity of NF-κB and its downstream proteins such as survivin, Bcl-xl, and XIAP, and affect the cell cycle (G2/M cell cycle arrest). These TQ analogs can further effectively inhibit the activity of the COX-1 and COX-2 enzymes, improve the activity of caspase-3, and release cytochrome c. In terms of sensitivity to oxaliplatin and GEM, the TQ analogs TQ-2G and TQ-5A1 have shown better effects than TQ-4A1 and are also better than the parent TQ at enhancing sensitivity to chemotherapeutic drugs (51). Another study found that TQ combined with D1T (a cationic liposome preparation of TQ) as the carrier could achieve a greater absorption rate, higher plasma concentration and bioavailability, smaller distribution volume, and easier clearance *in vivo*, which significantly improve its inhibitory effects on pancreatic tumors (52).

In order to improve the bioavailability of TQ to ensure that its therapeutic use is not limited to pancreatic cancer, several TQ carriers have also been developed. Due to the poor bioavailability and hydrophobicity of TQ, We summarized TQ in nanoparticles to improve its delivery (**Table 3**). The TQ-nanoparticle formulations showed improved anticancer and anti-inflammatory activities (66). To improve the clinical efficiency of TQ, researchers have formulated a colloidal carrier comprising TQ and solid lipid nanoparticles (SLNs). In the treatment of Huntington’s disease in rats, TQ-loaded solid lipid nanoparticles (TQ-SLNs) have exhibited stronger effects than TQ (53). Alam et al. also found that TQ-SLNs increased TQ delivery to brain tissues faster than free TQ (54). In addition, TQ-SLNs have stronger efficacy and better drug stability in the treatment of liver cirrhosis induced by paracetamol in contrast to the TQ-suspension (55). *In vivo* hepatoprotective investigations showed significant hepatoprotective effects for optimized TQ-self-nanoemulsifying drug delivery systems (TQ-SNEDDS) in comparison with TQ suspension. The relatively bioavailability of TQ was enhanced 3.87-fold by optimized SNEDDS in comparison with TQ suspension. In a male rat model, Elmowafy et al. found that TQ-NLCS (TQ-loaded nanostructured lipid carriers) had a stronger hepatoprotective effect compared to oral TQ (56). TQ-loaded phospholipid nanoconstructs (TQ-PNCs) have demonstrated significantly enhanced hepato-protective effects (57). TQ-NLCs have stronger antiproliferative activity and the ability to induce cell cycle arrest in breast and cervical cancers than TQ (58). TQ-NLCs can also improve the gastroprotective activity of TQ and protect against the formation of ethanol-induced ulcers (59). TQ-encapsulated nanoparticles (TQ-Nps) were synthesized using biodegradable, hydrophilic polymers such as polyvinylpyrrolidone (PVP) and polyethyleneglycol (PEG) to overcome the poor aqueous solubility, thermal and light sensitivity, and minimal systemic bioavailability of TQ, which can greatly improve the efficacy of cancer treatment (60). To exploit the benefits of the antioxidative property of TQ without any toxicity, Verma synthesized p-aminophenyl-1-thio-β-d-galactopyranoside (PAG)-coated N-isopropyl acrylamide (NIPAAM) nanoparticles followed by the encapsulation of TQ in their hydrophobic core. This method greatly improved the efficiency of TQ (61). TQ-loaded cyclodextrin nanoparticles were found to improve TQ solubility and its antiproliferative activity (62).TQ nanocapsules (actually containing half of the doses of TQ) produced better antihyperglycemic effect in type-2 diabetic rats as compared to TQ alone (63). Not only that, based on maximum concentration, time-to-maximum concentration, area-under-curve over 24 hours, and elimination rate constant, intranasal TQ-loaded nanoparticles (TQ-NP1) proved more effective in brain targeting compared to intravenous and intranasal TQ solution (64). The abovementioned studies have shown that the bioavailability of TQ can be significantly improved and its stability can be maintained using nanoparticles as TQ carriers. In addition, lipids can also act as TQ carriers. In a study on TQ and breast cancer, the encapsulation of TQ in liposomes was found to maintain its stability, improve its bioavailability, and enhance its anticancer activity (65). In order to improve the poor water solubility of TQ, TQ lipid spheres with a particle size lower than 70 nm were made. These lipid spheres result in deeper skin penetration, slow release, and skin compatibility, improving the anti-psoriasis effect of TQ (16).

**Table 3:** Mechanism of of thymoquinone carriers.

carriers	size	mechanisms	effects	references
D1T	110-120 nm	↑absorption, ↑plasma concentration, ↑bioavailability, ↑clearance, ↓distribution volume	↓pancreatic tumor	(52)
TQ-SLNs	none	↑efficiency, ↑ATPases function, ↓GFAP, ↓pro-inflammatory cytokines	↓Huntington’s disease	(53)
TQ-SLNs	188.66 ± 8.94 nm	↑affinity, ↑binding capacity, ↑5-HT, ↑DA, ↑NE	↑antidepressant activity	(54)
TQ-SLNs	66.1 ± 10.96 nm	↑bioavailability (5 fold), ↑stability, ↓SGOT, ↓SGPT, ↓ALP	↑liver function	(55)
TQ-NLCs	100~200 nm	↑bioavailability (3.97 fold), ↓ALT, ↓AST, ↓malondialdehyde, ↑GSH	↑liver function	(56)
TQ-PNCs	<100 nm	↑bioavailability (3.86 fold), ↓ALP, ↓ALT, ↓AST, ↓bilirubin	↑liver function	(57)
TQ-NLCs	35.66 ± 0.1235 nm	↑stability, ↑apoptosis, ↑cell cycle arrest, ↓proliferation	↓breast cancer	(58)
TQ-NLCs	75 ± 2.4 nm	↑pharmacokinetic	↑gastroprotective activity	(59)
TQ-Nps	<50 nm	↑efficiency, ↑anti-migratory, ↓toxic to normal cells, ↑miR-34a, ↓Rac1	↓breast cancer	(60)
NTQ	100 nm	↓toxic to normal cells, ↓AST, ↓ALT, ↓ALP, ↓LDH, ↓NF-kB, ↓COX-2	↑liver function	(61)
TQ-CD	400 nm	↓toxic to normal cells, ↑solubility, ↑antiproliferative	↓breast cancer	(62)
TQ-NCs	<100 nm	↑release profile, ↑efficiency	↑hypoglycemic effect	(63)
TQ-NP1	150 ~ 200 nm	↑drug-targeting potential, ↑efficiency	↓Alzheimer’s disease	(64)
TQ-LP	100 nm	↑bioavailability, ↓proliferation	↓breast cancer	(65)
TQ-LP	<70 nm	↑skin penetration, ↑release profile, ↑skin compatibility, ↓pro-inflammatory cytokines	↓psoriasis	(16)

TQ, thymoquinone; D1T, cationic liposomal formulation of thymoquinone; TQ-SLNs, thymoquinone-loaded solid lipid nanoparticles; TQ-NLCs, thymoquinone-loaded nanostructured lipid carriers; TQ-PNCs, thymoquinone-loaded phospholipid nanoconstructs; TQ-Nps, thymoquinone-encapsulated nanoparticles; NTQ, synthesized p-aminophenyl-1-thio-β-d-galactopyranoside-coated N-isopropyl acrylamide nanoparticles followed by the encapsulation of thymoquinone; TQ-CD, thymoquinone-loaded cyclodextrin nanoparticles; TQ-NCs, polymeric nanocapsules of thymoquinone; TQ-NP1, intranasal thymoquinone-loaded nanoparticles; TQ-LP, thymoquinone-loaded liposomes; GFAP, glial fibrillary acidic protein; NFκB, nuclear factor kappa-B; 5-HT, 5 hydroxytryptamine; DA, dopamine; NE, norepinephrine; ALT, alanine amino transferase; AST, aspartate amino transferase; GSH, hepatic reduced glutathione; SGOT, serum glutamic-oxaloacetic transaminase; SGPT, serum glutamic-pyruvic transaminase; ALP, alkaline phosphatase; LDH, lactic dehydrogenase; NF-κB, nuclear factor kappa-B; COX-2, cyclooxygenase-2; ↑, increase; ↓, decrease or inhibit.

## Discussion

The high mortality rate of pancreatic cancer and its resistance to chemotherapeutic drugs have long been major obstacles to medical treatment. Moreover, the surgical treatment of pancreatic cancer has reached a bottleneck without yet achieving ideal therapeutic effects. Finding a drug that can effectively control the development of pancreatic cancer is therefore crucial. Recent studies have highlighted TQ as a promising candidate in such therapies. In the past two decades, biologists have made remarkable progress in extracting effective ingredients, such as TQ, from plants to treat cancer. TQ is not harmful to normal human cells but has shown excellent antitumoral effects in a variety of cancers. Studies on pancreatic cancer have therefore attracted widespread attention (26, 67).

The mechanisms underlying the TQ-mediated apoptosis of pancreatic cancer cells currently include: the activation of the JNK and p38 MAPK pathways related to the downregulation of MUC4; ROS-mediated activation of the JNK pathway; cell cycle arrest; post-translational modification of histone H4 acetylation; and the activation of caspases. In a study by Torres (28), it was not directly confirmed whether TQ led to the activation of the TGF-β pathway and subsequent downregulation of MUC4. TGF-β was, however, determined to be a key molecule for the formation of the extracellular matrix and for the classic antifibrotic effects of TQ (68). The activation of TGF-β by TQ to downregulate MUC4 is theoretically possible but needs further confirmation. Relles et al. further confirmed TQ-mediated apoptosis of pancreatic cancer cells at the gene level (p53 and p31) and epigenetic level (H4 acetylated post-translational modification) (30). In contrast to genetic mutations that are almost impossible to reverse, epigenetic changes can be reversible and can therefore be receptive to drug intervention. HDAC inhibitors (HDAC-i) have been shown to exhibit antineoplastic activity in multiple tumor types, inhibit cell growth, and induce apoptosis (69). It is therefore possible that TQ may be used in clinics as an antitumoral drug. TQ can affect the cell cycle and promote the apoptosis of pancreatic cancer cells, which may be related to the lipophilicity of TQ facilitating its interaction with cells and subcellular structures (49). Surprisingly, there is an antagonistic relationship between juglone and TQ at low concentrations (33). TQ acts as an antioxidant (free radical scavenger) at low concentrations and as a pro-oxidant at high concentrations. Juglone-induced cytotoxicity in cancer cells often involves the production of ROS through redox activation (70, 71), which may comprise the possible underlying mechanisms of action that are responsible for the antagonistic effect between juglone and TQ.

The metastasis of pancreatic cancer is a multistep and multifactorial process that includes a series of changes in epithelial cell polarity, tumor cell adhesion, increased mobility, penetration of the basement membrane by cancer cells (72). A key step in metastasis involves the adherence of tumor cells to the extracellular matrix depending on focal adhesion and destroying the basement membrane to invade blood or lymphatic vessels. FAK is a key kinase involved in the formation of cell adhesive plaque. The downregulation of FAK expression can lead to changes in a wide range of signal-regulated proteins that are related to tumor invasion and migration, such as the downregulation of MMP-9 activity in the extracellular matrix, PI3K/Akt/NF-κB signal pathway inhibition, and F-actin depolymerization (73). In conclusion, the above-mentioned studies reveal that TQ can inhibit several key molecules such as FAK, Akt, NF-κB, and MMP-9 and that these molecules interact in a cascade to affect the metastasis of pancreatic cancer. TQ can therefore regulate the invasion and metastasis of pancreatic cancer cells at multiple levels with multiple targets.

We found that the main mechanisms of action of TQ involved in increasing chemosensitivity consist of blocking the Notch1/PTEN, PI3K/Akt/mTOR, and NF-κB signaling pathways, reducing PKM2 expression, and inhibiting the Warburg effect. Interestingly, the mTOR blocker everolimus can inhibit the Warburg effect by blocking the PI3K/Akt/mTOR signaling pathway, thereby reducing the sensitivity of pancreatic cancer cells to GEM (74). This suggests that TQ may also inhibit the Warburg effect by mediating the PI3K/Akt/mTOR signaling pathway, thus affecting the sensitivity of GEM, which needs further experimental confirmation. This also proves that the mechanisms of action of TQ involved in GEM sensitivity are both multitargeted and interrelated. In addition, when assessing the mechanisms underlying the promotion of apoptosis in pancreatic cancer, we found that TQ can affect the cell cycle and regulate Bax and Bcl-2. This indicates that TQ plays a synergistic role with GEM in promoting apoptosis. Notably, TQ combined with gemcitabine can increase the cell population in the G0–G1 phase. When combined with oxaliplatin, TQ can increase cell cycle arrest in the S phase. It is conceivable that TQ prevents the progression of the cell cycle through the M phase, which results from the inhibition of survivin and leads to apoptosis. However, studies on the causes of different arrest cycles of gemcitabine and oxaliplatin are limited. We speculate that different chemotherapeutic drugs may have different targets in the cell division cycle. In addition, TQ can coordinate with miRNA in increasing gemcitabine sensitivity (39, 40). Previous studies have found that TQ has the capacity to prevent tumors from progressing *via* regulating miRNAs, which in turn manage signaling pathways involved in the pathogenesis of cancer cells such as proliferation, metastasis, angiogenesis, apoptosis and epigenetic machinery (75). These results suggest that TQ can improve the chemotherapy sensitivity of pancreatic cancer at the epigenetic level.

MCP-1 is one of the key chemokines that trigger inflammation. It is also a key element in the immune response of malignant tumors. MCP-1 plays a dual role in the regulation of inflammation and immunity. Higher serum MCP-1 levels have been found to correlate with the increased macrophage infiltration of pancreatic cancer tissue and a favorable prognosis and overall survival rate (76). The level of MCP-1 in patients with pancreatic cancer and cachexia is also high (77). The level of MCP-1 has become one of the prognostic indicators of pancreatic cancer. The inhibitory effect of TQ on MCP-1 limits the occurrence and development of pancreatic cancer from both anti-inflammatory and immunomodulatory aspects.

If the size of a tumor exceeds 1–2 mm, it will require a continuous blood supply to maintain its growth. Once tumor cells have the ability to induce vascular growth, tumor growth becomes aggressive and facilitates tumor invasion and metastasis. Endothelial progenitor cells (EPCs), which are the precursors of a variety of endothelial cells, are the stem cells derived from the bone marrow that have the ability to proliferate and differentiate into mature endothelial cells. EPCs play an important role in tumor angiogenesis. TQ not only reduces the expression of VEGF at the molecular level but also directly ensures the inhibition of tumor angiogenesis *in vitro* (31, 43, 44). TQ may therefore be used as a new angiogenesis inhibitor in tumor therapy.

The redox properties of quinones can often trigger cancer cell apoptosis through oxidative stress induced by the in-situ production of ROS (78). c-Jun is both a pro- and anti-apoptotic protein depending on the cellular environment and other signals, and enables cells to deal with the damage caused by ROS production through the regulation of antioxidant genes (79). TQ affects pancreatic cancer calls not by inhibiting ROS and playing an antioxidative role for cancer prevention; rather, it induces ROS production causing oxidative stress reactions leading to the activation of the JNK pathway and in turn, pancreatic cancer cell apoptosis. In gliomas and colon cancer, TQ can play a role in promoting the oxidation of cancer cells by inducing ROS production (80, 81). The two seemingly opposite effects of TQ on the antioxidation of normal cells and pro-oxidation of cancer cells do not rule out that they are related to the specific structure of quinones (82).

Despite the promising anticancer effects of TQ, the main limitation for its clinical application lies in its hydrophobicity, poor bioavailability, sparing solubility, light- and pH-sensitive nature, and capacity to bind to plasma proteins, which prevents it from reaching its targeted tumor sites (83). To overcome these limitations, nanoparticle-based carriers have been developed, including polymeric, liposomal, and niosomal carriers, SLNs, and NLCs. Nanoparticles have many advantages, including enhanced biodegradability, controlled or sustained release, small size, and biocompatibility with tissues and cells, which make them the most suitable carriers for TQ. In addition, the encapsulation of TQ by nanoparticles provides the possibility of the fluorescent labeling of the compound, which would allow for the tracing of its route of entry, trafficking mechanisms, and intracellular distribution. In addition, the excipient used in the formulation of nano-TQ delivery systems should have a high degree of biocompatibility, biodegradability, and simple composition. Nano-TQ formulations usually contain a large amount of excipient, and therefore, the excipient should not have side effects (84). However, the formulation of nano-TQ delivery systems is a multistep process. At small scale, it is easy, but at the large scale, it becomes more difficult to control the batch-to-batch reproducibility. To overcome this problem, cold wet milling, spray drying, and a microfluidics system have been developed for efficient clinical translation of nanomedicines (85). Given these advantages, TQ-nanoparticle formulations may be more likely to be utilized in clinics than free TQ.

This review of the current status of research on TQ in relation to pancreatic cancer reveals that TQ can play an anticancer role by inhibiting the proliferation of cancer cells, promoting cell apoptosis, inhibiting invasion and metastasis, enhancing the sensitivity of chemotherapeutic drugs, and exhibiting anti-inflammatory effects. These anticancer mechanisms predominantly involve the NF-κB, PI3K/Akt, Notch, TGF-β, JNK, and p38 MAPK pathways, as well as the regulation of the cell cycle, which affects MMP-9 expression and PKM2 activity. Interestingly, the different anticancer mechanisms involve many similar associations. For example, TQ promotes apoptosis, inhibits invasion and metastasis, increases chemosensitivity, and exhibits anti-inflammatory mechanisms, which are all related to the inhibition of NF-κB and its downstream molecules. The mechanisms underlying the promotion of apoptosis and increasing GEM chemosensitivity are correlated with cell cycle arrest in the G0/G1 phases. Moreover, the inhibition of invasion, metastasis, and angiogenesis is related to the regulation of MMP-9 and VEGF. Thus, the anticancer effects of TQ on pancreatic cancer are multidirectional but interrelated. It has further been confirmed that TQ is a promising multitarget anticancer drug with a variety of molecular mechanisms of action that underlie its multidirectional inhibition of tumor development. The synergistic effect of TQ on drugs may also contribute to resolving drug resistance. At present, studies on TQ and pancreatic cancer are mainly limited to laboratory research on the molecular mechanisms involved; however, the discovery of TQ carriers is paving the way for its clinical applications. We expect that the research on TQ will move from the experimental stage to clinical trials in the near future.

## Author contributions

ZZ contributed to manuscript drafting; LL handled the revision of the manuscript for important intellectual content; SL contributed to pharmaceutical guidance; XH contributed to picture processing; JY contributed to article review. ZZ, LL, SL, and XH contributed equally to this work. All authors have agreed to be accountable for the content of the work.
